# Effect of group psychotherapy on the annual incidence of self-harm and suicide attempts in borderline personality disorder: A pilot study

**DOI:** 10.1192/j.eurpsy.2021.468

**Published:** 2021-08-13

**Authors:** I. Figuereo, J. Labad, A. González-Rodríguez, M. Santamaria, A.I. Cebrià, D. Palao Vidal, J.A. Monreal

**Affiliations:** 1 Mental Health., Parc Tauli University Hospital. I3PT. UAB, CIBERSAM, Sabadell, Spain; 2 Mental Health, Hospital of Mataró. Consorci Sanitari del Maresme. CIBERSAM., Mataró, Spain; 3 Mental Health, Parc Taulí University Hospital. Autonomous University of Barcelona (UAB). I3PT, Sabadell, Spain; 4 Mental Health., Hospital Universitari Mútua de Terrassa. CIBERSAM, Terrassa, Spain

**Keywords:** Borderline personality disorder, psychotherapy, Suicide, Self-harm behavior

## Abstract

**Introduction:**

Borderline personality disorder (BPD) has been characterized by mood instability, impulsive behavior and eventual dissociative and psychotic symptoms. Around 70% of patients present repeated self-injury behavior which is associated with high risk of completed suicide.

**Objectives:**

To investigate the effect of group psychotherapy on the annual incidence of self-harm behavior and suicide attempts in BPD.

**Methods:**

We carried out a retrospective longitudinal study by selecting BPD patients who received group psychotherapy during 2016. Systems Training for Emotional Predictability and Problem Solving (STEPPS) or Mentalization-Based Treatment (MBT) psychotherapies were applied. Patients without any self-harm/suicidal attempt before the intervention, those with comorbid diagnosis and those who did not engage at least half of total sessions were excluded for final analyses. Number of self-harm events, suicide attempts and other clinical events were recorded and compared one-year before and one-year post-intervention. SPSS software version 21.0 (IBM) was used for statistical analyses. Nonparametric tests and Survival tests were performed.

**Results:**

Eight women out of 35 fulfilled our inclusion criteria. After group psychotherapy, a significant reduction in the number of self-harm events and suicidal attempts was found (mean 1.9+/-1.4 vs 0.5+/-1.1; p=0.042). Survival tests revealed significant differences in the occurrence of suicidal attempts. We did not find significant differences in the other clinical events.

**Conclusions:**

Our results show a clear effectiveness of group psychotherapy in reducing self-harm events and/or suicidal attempts in BPD patients. If these findings are confirmed in future studies including larger samples, group psychotherapy could be indicated for diminishing suicide risks in BPD.

**Disclosure:**

No significant relationships.

